# Modelling spatio-temporal patterns of disease for spatially misaligned data: An application on measles incidence data in Namibia from 2005-2014

**DOI:** 10.1371/journal.pone.0201700

**Published:** 2018-08-13

**Authors:** D. Ntirampeba, I. Neema, L. Kazembe

**Affiliations:** 1 Department of Mathematics and Statistics, Polytechnic of Namibia, Windhoek, Namibia; 2 Namibia Statistics Agency (NSA), Windhoek, Namibia; 3 Department of Statistics and Population Studies, University of Namibia, Pionerspark, Windhoek, Namibia; Sciensano, BELGIUM

## Abstract

**Background:**

Making inferences about measles distribution patterns at small area level is vital for more focal targeted intervention. However, in statistical literature, the analysis of originally collected data on one resolution with the purpose to make inferences on a different level of spatial resolution is referred to as the misalignment problem. In Namibia the measles data were available in aggregated format at regional level for the period 2005 to 2014. This leads to a spatial misalignment problem if the purpose is to make decisions at constituency level. Moreover, although data on risk covariates of measles could be obtained at constituency level, they were not available each year between 2005 and 2014. Thus, assuming that covariates were constant through the study period would induce measurement errors which might have effects on the analysis results. This paper presents a spatio-temporal model through a multi-step approach in order to deal with misalignment and measurement error.

**Methods:**

For the period 2005–2014, measles data from the Ministry of Health and Social Services (MoHSS) were analysed in two steps. First, a multi-step approach was applied to correct spatial misalignment in the data. Second, a classical measurement error model was fitted to account for measurement errors. The time effects were specified using a nonparametric formulation for the linear trend through first order random walk. An interaction between area and time was modelled through type I and type II interaction structures.

**Results:**

The study showed that there was high variation in measles risk across constituencies and as well as over the study period (2005–2014). Furthermore, the risk of measles was found to be associated with (i) the number of people aged between 0 and 24 years, (ii) the percentages of women aged 15–49 with an educational level more than secondary, (iii) the percentages of children age 12–23 months who received measles vaccine, (iv) the percentages of malnourished children under 5 years, and (vi) the measles cases for each previous year.

**Conclusion:**

The study showed some of the determinants of measles risk and revealed areas at high risk through disease mapping. Additionally, the study showed a non-linear temporal distribution of measles risk over the study period. Finally, it was shown that ignoring the measurement errors may yield misleading results. It was recommended that group and geographically targeted intervention, prevention and control strategies can be tailored on the basis these findings.

## Introduction

Measles is among the most transmissible of human infections, caused by a virus which is a member of genus *Morbillivirus* of the family of *Paramyxoviridae* [1] and it is known to infect any persons, via airborne droplet, who have not previously had the disease or been successfully immunized [2]. It has an incubation period of 7 to 18 days from exposure to onset of fever [2]. Although the measles vaccine has been available for the past five decades, measles has remained one of the leading causes of death among children, especially in developing countries with low capita per incomes and poor health service systems [2, 3]. In communities and areas where the immunization is not widely spread, more than 90% of people are infected by the age of 20. Because there is no antiviral treatment for measles virus, vaccination and supportive care, such as good nutrition and adequate fluid intake, are mainly used to fight measles [4].

The goal of elimination of measles has been reached in countries of the World Health Organization (WHO)Region of the Americas through measles vaccine and careful measles surveillance. In other WHO regions, the complete elimination goals of measles were set for 2012 and 2015 in Western Pacific and European and Eastern Mediterranean regions, respectively, whereas Africa and South-East Asia have set their target for 2020 [2]. Consequently, measles cases are still reported in many countries [5]. Various studies have shown that the distribution of measles risks vary quite often spatially due to different risk factors such as level of immunization, susceptible population, and other indicators, many socio-economic [6, 7]. Maps resulting from spatio-temporal analysis of variations in measles incidences are often used to identify changes over time and areas of a region or a country with most disease occurrences in order to plan for a proper intervention and targeted distribution of aid to most affected areas [7]. They are indeed regarded as useful tools for geographically targeted interventions, monitoring, and evaluation of infectious diseases such as measles. However, because of confidentiality issues, spatio-temporal analyses of disease surveillance data, such as measles data, are often presented in aggregated form over time or an area. Nevertheless, health decisions might be needed at lower administrative boundaries from the levels where data were originally collected.

Direct inferences at such levels, which are made on basis of the original level of aggregation, lead to complications known as a misalignment problem in the statistical literature [8]. Moreover, many researchers do not account for measurement error, despite the awareness of its presence and potential effects on analysis results [9]. Such studies assume that surrogate variables are the same as the variables of interest. Research has shown that ignoring measurement error may, for example, lead to masking some important features of data, losing power of hypothesis testing of relationships among variables, and introducing bias in estimates [10].

In this study, we used measles incidence data aggregated to regional level in Namibia during 2005–2014 to fit spatio-temporal models, which would help to identify constituencies (lower level of regions) at high risk, as well as to visualise smoothed patterns of measles risk. Furthermore, the study aimed to determine factors associated with the distribution and the dynamics of measles in Namibia while accounting for measurement error that might be present in the covariates.

## Methods

### Settings

**Fig 1:**
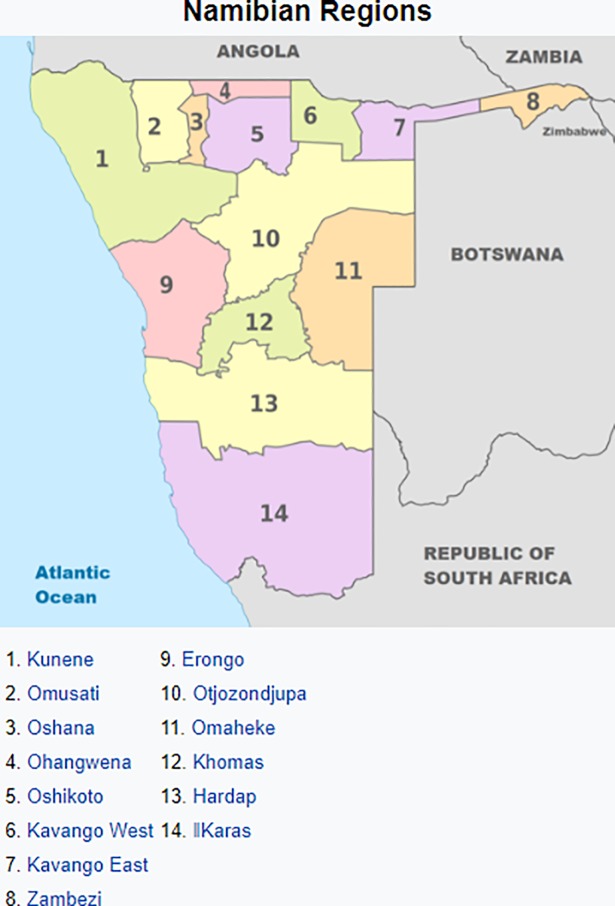
Namibian regions (Source:https://en.wikipedia.org/wiki/Regions_of_Namibia).

Each of the 14 administrative regions in Namibia is sub-divided into constituencies, giving a total of 121 sub-regions (i.e. constituencies). Fig 1 shows the map of Namibia with14 regions and its neighbouring countries.

From the 2011 Namibia population and housing census (NPHC), the Namibia population stands at 2,113,077. Due to presence of the arid Namib Desert, the population densities vary substantially among the regions, with more than two-thirds of the population estimated to live in the northern regions, whereas less than one-tenth lives in the south.

### Data

The Ministry of Health and Social Services (MoHSS) has granted permission to use measles data. Additionally, measles data were accessed anonymously.

Data on reported measles cases over contiguous regions in Namibia are available from the Ministry of Health and Social Services (MoHSS) database for the period 2001 to 2014. Due to improvement of the Namibian surveillance health system, the period of 2005–2014 provided consistent information for the entire country, and hence only data from this period were considered in this study. The database included all suspected measles cases from which confirmed cases were extracted. A suspected case is any person with fever and maculopapular generalised rash, and cough or red eyes. A confirmed case is any suspected case with laboratory confirmation or epidemiological links to confirmed cases in any outbreak [2].

In this study, the determination of a measles case followed the WHO standard definition, which considers a measles case as either a clinically-confirmed case or an epidemiological linked case or a laboratory confirmed case [2].

The following variables, as identified through literature [6, 7, 11, 12, 13], were considered as covariates in the model, each measured at constituency level.

- Number of people aged between 0 and 24 years, which represents the proxy of the size of susceptible group [6] for each constituency (LST)
- Employment rates (EmployR)
- Percentages of children aged 12–23 months who received measles vaccine (Vaccination coverage (Vacc))
- Educational attainment of female household population (percentages of women aged 15–49 years with an educational level more than secondary) (Edu)
- Percentages of malnourished children under 5 years (Malnou)
- Measles cases for each previous year were treated as a determinant factor of the measles count in the subsequent year (PrevCase)

Table 1 gives the description of the variables used for this study. Administrative boundary maps were obtained from the Namibia Statistics Agency (NSA) head office.

**Table 1:** Description of variables considered for the analysis.

Description	Min, Max	Source
Number of people aged between 0 and 24 years	2691; 26605	2011 NPHC
Employment rates	28; 92.9	2011 NPHC
Percentages of children aged 12–23 months who received measles vaccine (Vaccination coverage)	75; 98.7	2013 Namibia Demographic Health Survey (NDHS)
Percentages of women aged 15–49 years with an educational level more than secondary	2.7; 24.4	2013 NDHS
Percentages of children under 5 years classified as malnourished according to anthropometric index of nutritional status (weight-for age: % below -3SD)	0.9; 5.7	2013 NDHS
Counts of measles for previous year	0; 207	MoHSS

### Statistical methods

The counts of measles cases were available at regional level, and the aim of this study is to estimate the relative risk of measles at constituency level. In statistical literature, the analysis of originally collected data on one resolution with the purpose to make inferences on a different level of spatial resolution is referred to as the misalignment problem [14]. Thus, if the data are to be analyzed at regional level with the purpose of making decisions at constituency level, a misalignment is introduced in the analysis. To overcome misalignment, a multi-step approach, discussed in Ntirampeba *et al*. [15], was used. Briefly, the multi-step approach fundamentally involves two steps. First, a total count of measles cases for constituency *i* is computed using the population proportional allocation of cases. Thereafter, the hierarchical smoothing techniques are used to estimate the relative risk of measles. In this study, the number of measles cases in the constituency *i* of region *k* in year *j* was computed as $y_{ikj}=\frac{P_{ikj}}{P_{kj}}Y_{kj}$, where *Y*_*kj*_ is the number of measles cases in the k^th^ region for year *j* that contains the constituency *i*; *P*_*ikj*_ is the total population of constituency *i* in the region *k* during year *j*; and *P*_*kj*_ is the total population of the region *k* in year *j*.

A Poisson hierarchical regression model was used to estimate the spatial and temporal dynamic of measles in Namibia. Thus, the following distribution for the computed measles cases was specified:

$$y_{ikj}|m_{ikj}∼Poisson(E_{ikj}m_{ikj}),$$ (1)

where *E*_*ikj*_ is the expected number of disease cases for constituency *i* in the k^th^ region for year *j*. *λ* is assumed to be the 2014 Namibia annualised measles incidence per 100,000 inhabitants such that *E*_*ikj*_ = *λP*_*ikj*_, and *m*_*ikj*_ is the relative risk for contracting the disease in the constituency *i* of region *k* during the year *j*.

The focus in the analysis is on the form of the regression model for the log relative risk *m*_*ikj*_, which is specified as a function of fixed effects (i.e. covariates, where some of the covariates might not be directly observed, in the constituency *i* for year *j*), spatial random effects, temporal effects, and spatio-temporal interaction effects.

#### Fixed effects modelling

The fixed effects were modeled as a linear combination of covariates available in constituencies for each year. That is $X_{ikj}^{T}\beta$, where for fixed effect parameters *β*, a weakly informative Gaussian prior $\beta ∼N(0,\tau_{\beta }^{‑1}I)$ with small precision $\tau_{\beta }(i.e.\;\tau_{\beta }^{2}=0.0001)$ on identity matrix was assumed. By the rule of thumb, a covariate is significantly associated with the measles risk if the credible interval (CI) corresponding to its fixed effects does not contain zero.

#### Spatial random effects modelling

The spatial trends were modelled as a sum of constituencies’ heterogeneities and spatial clustering effects. For the unstructured spatial random effects, ∅_*ikj*_, an independent and identically distributed (IID) latent model was assumed such that is ∅_*ikj*_ ∼ *N*(0,1/*τ*_∅_). These spatial random effects control globally the extra- variability in log relative risks. Under this model, the effect ∅_*ikj*_ for each constituency is independent of all other constituencies. For the structured spatial random effects, *ω*_*ikj*_, we assumed a Besag-York-Mollie specification [16] such that *ω*_*ikj*_ is modeled using an intrinsic conditional autoregressive structure model (ICAR).

$$\omega_{ikj}|\omega_{\dot{i}kj\neq ikj}∼N\left(\frac{1}{N_{i}}\sum_{i}\omega_{ikj},1/\tau_{w_{i}}\right),$$ (2)

where $\tau_{w_{i}}$ and *N*_*i*_ are the precision parameter and the number of neighbours of constituency *i* respectively. Under this latent model, the effect of *ω*_*ikj*_ for each constituency *i* is normally distributed with mean effect equals the average of effects of neighbours of constituency *i* and $\tau_{w_{i}}$ precision. With this model, the adjacency matrix was used to characterise the spatial relationships between constituencies. The neighbours are defined in terms of constituencies sharing at least one point (queen adjacency) and the weight is set to be one if two constituencies are neighbours, otherwise the weight equals zero [16]. The priors for the precisions of both unstructured and structured spatial random effects were assumed to follow inverse gamma distributions ($\tau_{\emptyset }^{2}∼IG(0.0001,0.0001)$ and $\tau_{w_{i}}^{2}∼IG(0.0001,0.0001)$).

#### Temporal and time-space interaction effects modelling

The time effects can be modeled using time as a categorical variable through the introduction of dummy variables; using parametric linear trend or using nonparametric formulations to relax the assumption of linear trends through random walk models [17, 18]. In this study, we opted to specify the time effects using a nonparametric formulation for the linear trend through first order random walk and a Gaussian exchangeable latent model.

$$\gamma_{t}|\gamma_{t-1}∼N(\gamma_{t+1},\sigma_{\gamma }^{2}),\;\mathrm{for}\;t=1,$$ (3)

$$\mathrm{and}\;\theta_{t}∼N(0,1/\tau_{\theta }),$$ (4)

where *γ*_*t*_ and *θ*_*t*_ represent structured (through neighborhood structure) and unstructured temporal effects, respectively. An interaction between area and time is modeled by expanding the temporal effects through addition of an interaction term (*δ*_*it*_). This interaction term explains the differences in time trend for different areas (i.e. constituencies). There exist various specifications for this term [18, 19]:

Type I interaction assumes that the two unstructured effects ∅_*ikj*_ (spatial effect) and *θ*_*t*_ (temporal effect) interact. The structure matrix for this type is expressed as follows. *R*_*δ*_ = *R*_∅_⨂*R*_*θ*_ = *I*⨂*I* = *I*. Since both ∅_*ikj*_ and *θ*_*t*_ do not have a spatial or temporal structure, an identically independent non-informative normal model for *δ*_*it*_ is used.

$$\mathrm{That}\;\mathrm{is}\;{\delta_{it}∼N(0,\sigma_{\delta }^{2})\;\mathrm{with}\;\sigma }_{\delta }^{2}=0.0001.$$ (5)

In type II interaction, the structured temporal main effect *γ*_*t*_ and the unstructured spatial effect ∅_*ikj*_ interact. The interaction structure matrix is given by

$$R_{\delta }=R_{\emptyset }⨂R_{\gamma },$$ (6)

where *R*_∅_ = *I* and *R*_*γ*_ is a neighborhood structure that may be defined through a random walk. Thus, a random walk across time for each area independently from all other areas is assumed for *δ*_*it*_. This implies i^th^ element (i.e. i^th^ area parameter) of the vector parameter {*δ*_*i*1_,…,*δ*_*iT*_} has an autoregressive structure on the time component, which is independent from the ones of other elements.

For type III interaction, the interacting parameters are the unstructured temporal effect *θ*_*t*_ and the structured main spatial effect *ω*_*ikj*_. The structure matrix is written as

$$R_{\delta }=R_{\theta }⨂R_{\omega },$$ (7)

where *R*_*θ*_ = *I* and *R*_*ω*_ is a CAR neighborhood structure. Thus, the parameters of the i^th^ time point {*δ*_*i*1_,…,*δ*_*iT*_} have a spatial structure independent from the other points.

The type IV interaction structure combines spatial and temporal structured effects (i.e. *ω*_*ikj*_ and *γ*_*t*_). The resulting interaction matrix can be written as

$$R_{\delta }=R_{\theta }⨂R_{\omega }.$$ (8)

This is believed to be the most complex interaction structure [20]. It has been argued that the identifiability of highly structured interaction, such as type III and type IV, from the main effects may be doubtful [21]. Therefore, this study will explore only the type I and type II interaction structures.

By combining fixed effects, spatial effects, temporal effects, and space-time interaction effects together, we obtained the regression model for the log relative risk as shown:

$$log(m_{ikj})={\log(E_{ikj})+X}_{ikj}^{T}\beta +\emptyset_{ikj}+\omega_{ikj}+\gamma_{t}+\theta_{t}+\delta_{it}.$$ (9)

#### Measurement error models

Fundamentally, the specification of a measurement error model is based on an assumption about the distribution of the observed values given the true values or vice versa [9]. For the classical measurement error model, the distribution of the observed values given the true values is specified, while for the latter specification is referred to as Berkson error model. That is, the classical measurement error model is expressed as (*W* = *w*|*x*), while the Berkson error model is given by *P*(*X* = *x*|*w*), where *X* and *W* are the true and observed covariates, respectively. In this study, the errors in covariates were modeled using an additive non-differential classical measurement error model with respect to the response variable. In other words, the measurement error model does not depend on the value of the response variable and *w*|*x* = *x* + *u*. In this case, *w* are observed values of the true but unobserved covariates *X* (i.e. *W*s are surrogate of *Xs*). The error term *u* assumed a Gaussian prior with a zero mean and a covariance matrix *C* = *τ*_*u*_*D* (i.e. *u* ∼ *N*(0,*C*)), where $\tau_{u}\;(\tau_{u}^{2}∼loggamma(1,0.0005))$ is the precision of the error term and *D* is a diagonal matrix of fixed scaling values (*d*_*i*_) of the observational precision.

By including the error model in the Eq (9), the regression model for the log relative risk becomes

$$log(m_{ikj})={\log\left(E_{ikj}\right)+X}_{ikj}^{T}\beta +W_{ikj}^{T}\beta^{*}+\emptyset_{ikj}+\omega_{ikj}+\gamma_{t}+\theta_{t}+\delta_{it},$$ (10)

where $W_{ikj}^{T}=X_{ikj}^{*T}+u$ is a vector of adjusted mismeasured covariates obtained by applying classical measurement error modelling on mismeasured covariates ${(X}_{ikj}^{*T})$, β* is the vector of parameters corresponding to $W_{ikj}^{T}$. Details on measurement error models can be found elsewhere [9, 22].

#### Analysis of measles data

A preliminary descriptive analysis of confirmed measles cases was performed to gain insight about the shifts of measles yearly incidence (Fig 1). Poisson models (Table 2) were built in Bayesian modelling framework using R-INLA (R-version 3.4.3, Integrated Nested Laplace Approximation -INLA). The first three models (Table 2) assumed spatial random components as the only sources of variability in the risk of measles. In these models, unstructured and structured random effects were considered. For unstructured random effects model (Model 1), the spatial trend includes IID random effects. Two models for structured random effect for constituencies were considered. Model 2 assumes for each region a spatial random effect that is distributed as a function of the mean effect of regions in neighbourhood (ICAR). Model 3 is convolution model that assumes for each region two components of random effect, namely, specific region random effect (specific region heterogeneity) and structured random effect (random effect due to clustering). These models were extended by adding covariates and spatio-temporal component in parametric formulation fashion that assumes linearity in the global time effect and the differential trend for constituency and time (Models 4–6). To relax the assumption of linearity in constituency-time component, a non-parametric modelling approach (model 7–14 in Table 2) was employed. For space-time interaction, this study explored only the type I and type II interaction structures.

**Table 2:** Summary of models fitted to measles data for Namibia and their corresponding deviance information criterion (DIC) and sum of log of conditional predictive ordinate (SumLog(CPO)). ICAR—intrinsic conditional autoregressive structure model, CAR—Conditional Autoregressive, IID—identically distributed.

Poisson model	Spatial component	Fixed effect	D	p	DIC	SumLog(CPO)
1	ICAR	None	14617.09	94.73	14806.55	-7558.995
2	CAR	None	14616.81	94.96	14806.73	-7559.032
3	IID	None	14613.48	99.71	14812.9	-7577.505
4	ICAR	All covariates (parametric model)	13173.53	152.61	13478.75	-7104.421
5	CAR	All covariates (parametric model)	13173.23	152.76	13478.75	-7104.024
6	IID	All covariates (parametric model)	13170.76	166.18	13503.12	-7166.587
7	ICAR	All covariates (non-parametric model)	7532.17	104.02	7740.21	-4297.502
8	CAR	All covariates (non-parametric model)	7531.79	104.20	7740.19	-4298.066
9	CAR	All covariates +time-space interaction (Type I)	3765.80	617.93	5001.66	-2853.179
10	ICAR	All covariates + time-space interaction (Type I)	3765.58	618.01	5001.60	-2852.438
11	CAR	All covariates + time-space interaction (Type I) + measurement error models	3765.35	617.16	4999.69	-2850.238
12	ICAR	All covariates + time-space interaction (Type I) + measurement error model	3748.53	629.33	5007.19	-2893.197
13	CAR	Covariates + time-space interaction (Type II) + measurement error models	3766.41	617.16	5000.73	-2835.843
14	ICAR	Covariates + time-space interaction (Type II) + measurement error models	3782.19	608.52	4999.23	-2831.314

In addition, error models were used for some variables (i.e. Percentages of children under age 5 classified as malnourished according to anthropometric index of nutritional status (weight-for age: % below -3SD); and Employment rates) in order to correct for mismeasuring. All models fitted in this study are summarised in Table 2.

The best model was selected using the combination of the deviance information criterion (DIC) given by *DIC* = *D* + 2*p*, where *D* is the deviance evaluated at the posterior mean and *p* the effective number of parameters in the model, and the statistic ∑log(CPO) (where CPO is a conditional predictive ordinate). The rule of thumb indicates that the best model is one with the smallest value of DIC and a larger value of ∑log(CPO) denotes a better fitting. The summary statistics of the best model is presented in Table 3.

**Table 3:** Summary statistics: Mean, standard deviation and 95% credible interval of the posterior distribution of the fixed effects for Model 11.

Variable	Mean	Std dev	95% CI
Percent of women aged 15–49 with an educational level more than secondary	-0.0646	0.0145	-0.0935, -0.0366
Counts of measles for previous year	0.1020	0.0440	0.0178, 0.1908
Employment rates	-0.0561	0.0023	-0.0616, -0.0513
Number of people aged between 0 and 24 years	0.8487	0.0727	0.7081, 0.9934
Percentages of children age 12–23 months who received measles vaccine	-0.0379	0.0147	-0.0677, -0.0103
Percent of children under age 5 classified as malnourished	0.0643	0.0285	0.0019, 0.1128

## Results

### Exploratory results

**Fig 2:**
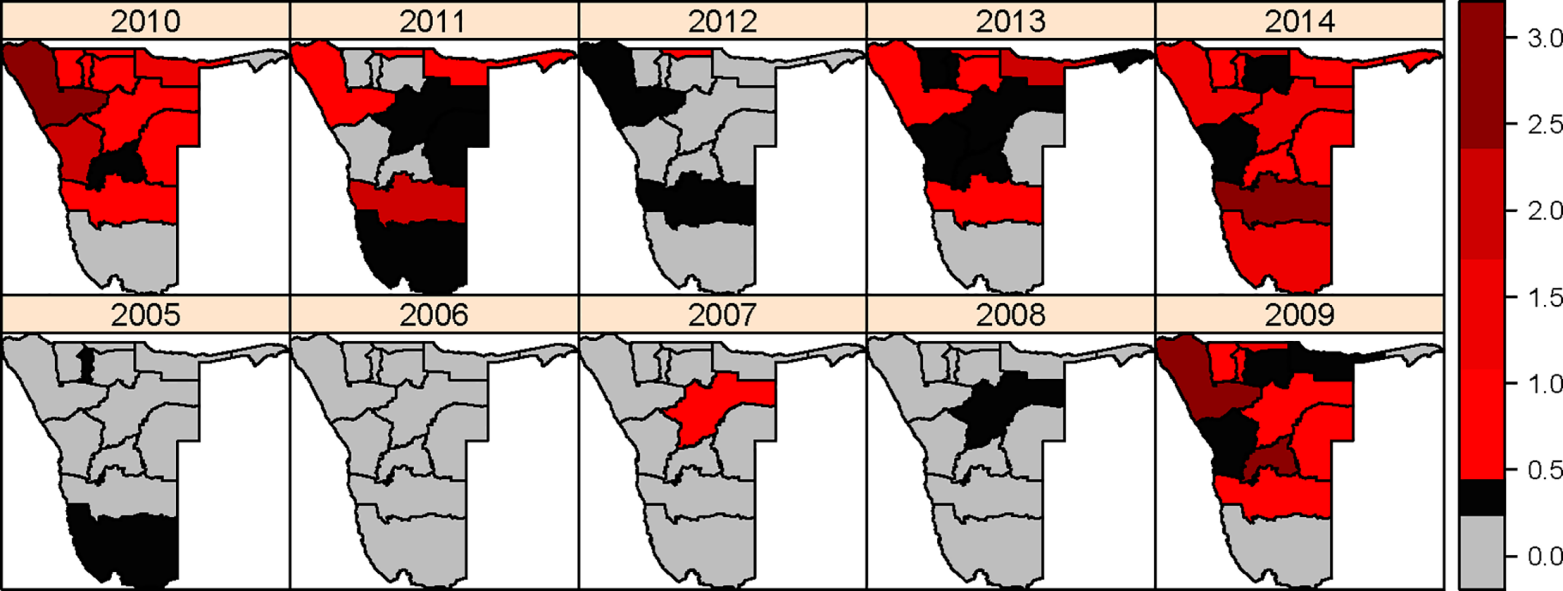
Measles incidence rates (number of cases per 1000 inhabitants) in Namibia for during 2005–2014.

**Fig 3:**
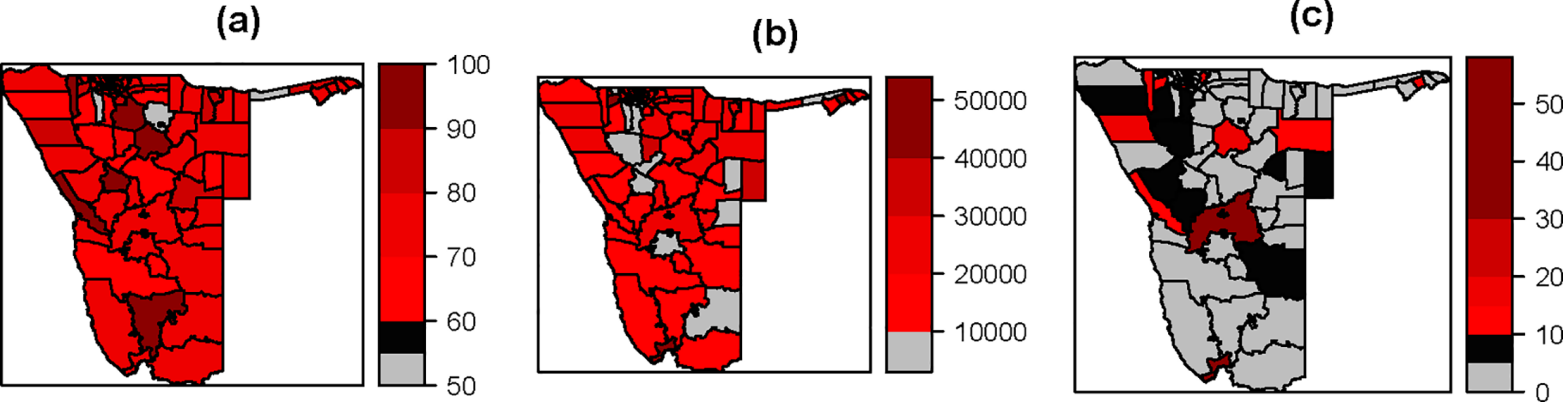
Maps of some of the key input variables; (a) map of vaccination coverage at constituency level (in percentage, obtained from 2013 Demographic Health Survey (DHS)), (b) map of population at constituency level (obtained from 2011 Population and Housing Census), and (c) map of percentage of women aged 15–49 with an educational level more than secondary (2013 DHS).

A total of 9923 cases were recorded for the period 2005–2014 in the 13 administrative regions in Namibia. Fig 2 presents the spatio-temporal distribution of measles incidence rates in Namibia for the period 2005–2014. The regional distributions of measles incidence rates for each region are shown. Fig 2 shows a great variation in measles occurrence over the 13 regions. In four regions, namely Kavango, Khomas, Kunene, and Ohangwena, high measles incidence rates were observed through the study period. Fig 3(A) shows that constituency in Kunene and Khomas regions had vaccination coverage between 60% and 80%, whereas for constituencies in Ohangwena and Kavango regions the vaccination coverage was between 50% and 80%. From Fig 3(B), it was observed that most of the constituencies in Okavango, Kunene, Khomas, and Ohangwena are highly populated (i.e. between 30000 and 50000 inhabitants). Fig 3(C) indicates that most of the constituencies had low percentages of women aged between 15–49 years with an educational level more than secondary. It also shows that constituencies in the northern part of Namibia had less than 10% of women aged between 15–49 years had attained more than secondary education level.

### Fixed effects

The fixed effects allow identifying which covariates are significantly associated with an increase or decrease in measles risks. Consequently, the public health efforts can be directed towards improving and or reinforcing areas in which health care is more needed.

**Fig 4:**
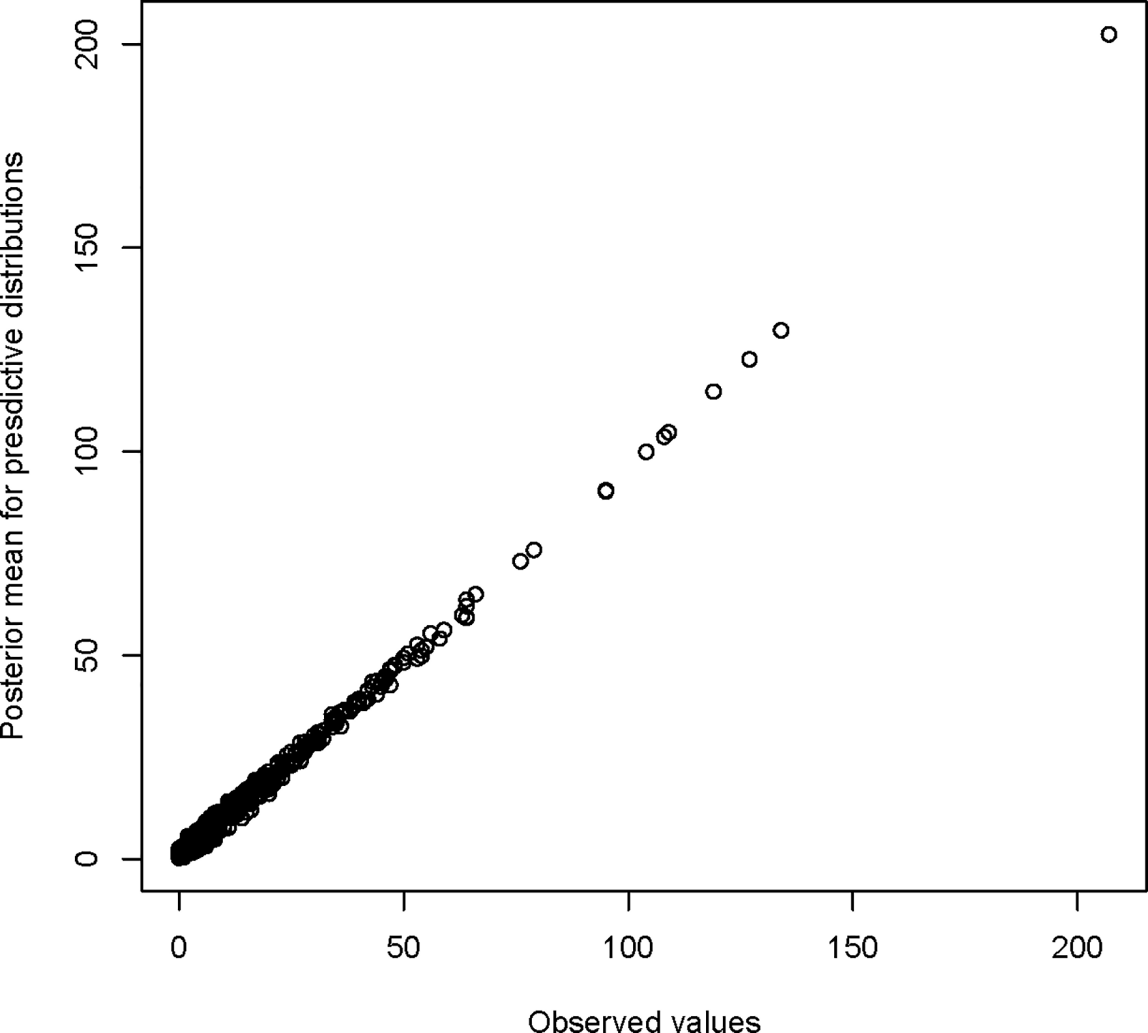
Scatterplot of the posterior mean for the predictive distributions against observed values (measles cases).

**Fig 5:**
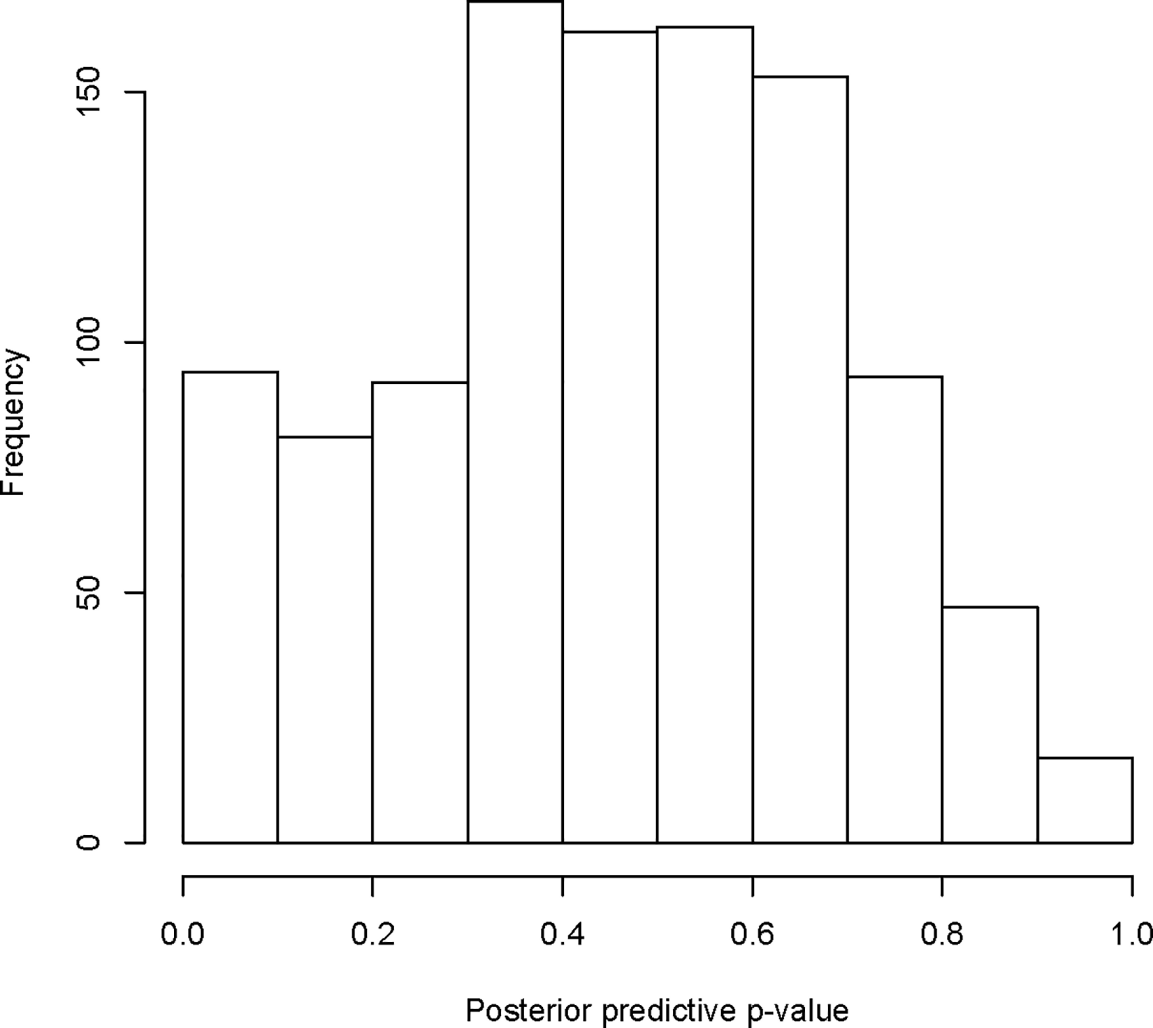
Histogram of the posterior predictive p-values.

Based on DIC values, Model 9, which included all covariates, unstructured and structured random effects, and time-area interaction term (Type I), emerged the best fit among fitted naïve models for this data (Table 2). By including error models in covariates, “the number of people aged between 0 and 24 years (LST)” and “the percent of children under 5 years classified as malnourished”, the model (Model 11, 12, and 13) performed relatively better than Model 9. However, these three models performed equally well as their DIC values were almost similar. To compare these competitive models in terms of the prediction performance, the statistic ∑*log*(*CPO*) is commonly used [17]. Larger value of the quantity indicates better model fit. From Table 2, it can be noted that Model 11 has a larger value of ∑*log*(*CPO*). Fig 4 shows that the predictions are very close to the observed values. Also, it can be noted from Fig 5 that most of the p-values are in the middle range, while very few p-values are at either side of the distribution, which suggests again that the model fits data rationally well. Therefore, this model represents a better fitting. Thus, we presented in Table 3 a summary of results of Model 11 only. Based on the 95% credible interval, the percent of women aged 15–49 years with an educational level more than secondary (Edu), the number of people aged between 0 and 24 years (LST), percentages of children aged 12–23 months who received measles vaccine (Vacc), and the counts of measles for previous year (PrevCase), the employment rates, and the percentages of children under 5 years classified as malnourished had significant effects on measles risks. For example, an increase of 1% in the percent of women aged 15–49 years with an educational level more than secondary implies a decrease of approximately 6% in the risk of measles. Also, an increase in 1 unit the log of the number of people aged between 0 and 24 years is associated with an increase of around 133.7% in the risk of measles.

### Spatio-temporal distribution of measles relative risks

**Fig 6:**
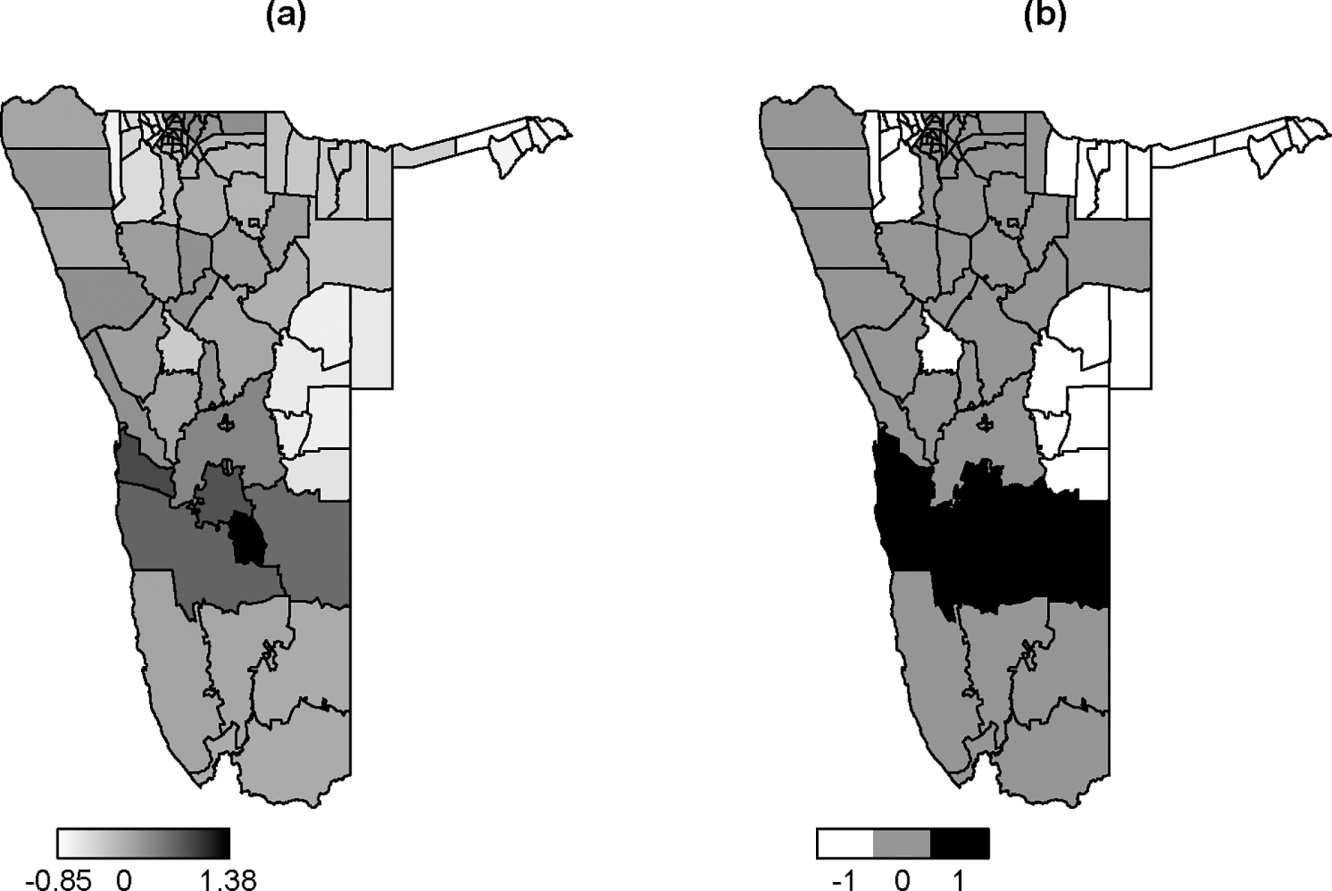
Distribution of spatial random effects (Model 11); (a) map of means of the posterior distribution of the spatial structured random effect (in Model 11), (b) Map of significant means of the posterior distribution of the spatial structured random effect in Model 11.

Fig 6 shows the maps of the distribution of posterior means of structured random effects and the significant observed structured random effects. From Fig 6(A), constituencies in Omusati, Caprivi, Omaheke, and Omaruru regions were negatively associated with measles risks (i.e. they have negative random effects). For Fig 6(B), three different numbers were used to distinguish significant observed random effects. White denoted by (-1) indicated significant negative random effects, grey denoted (0) indicated non-significant random effects, and black denoted by (1) represented significant positive random effects. From this figure, it was confirmed that the constituencies in Omusati, Caprivi, Omaheke, part of Kavango, and Omaruru regions had significant negative random effects on measles. In order words, the constituencies in those regions were inversely significantly associated with measles risks.

**Fig 7:**
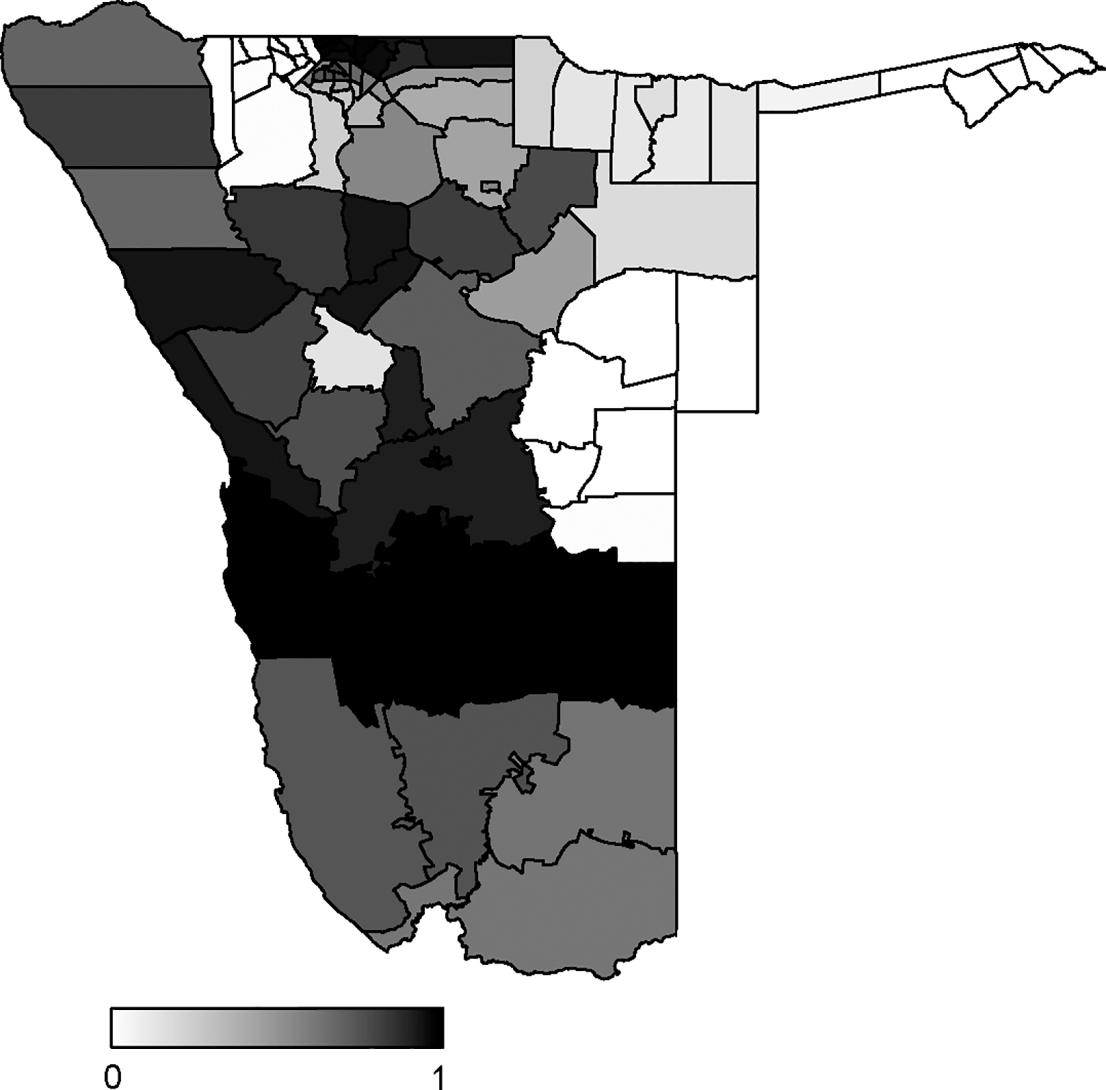
Distribution of constituency specific posterior probabilities *p*(*SRR* > 1|*y*) in Model 11. SRR: specific relative risk.

Fig 7 shows the distribution of posterior probabilities of constituencies with specific relative risks (SRR) exceeding one after adjusting for covariates. In Kunene region, the Opuwo, Khorixas, and Outjo constituencies had higher measles risks. There was a higher measles risk in most of constituencies in Ohangwena region. In Otjozondjupa region, Okahandja and Otjowarongo constituencies had moderate probabilities to be classified as areas at high risk of measles. For Khomas regions, the constituencies in Windhoek Urban had very high measles risk compared to Windhoek rural constituency. All Hardap region’s constituencies had high probabilities of relative risks exceeding one. The probability that the measles relative risk exceeds one was close to zero for the constituencies in Omusati, Omaheke, and Caprivi regions.

**Fig 8:**
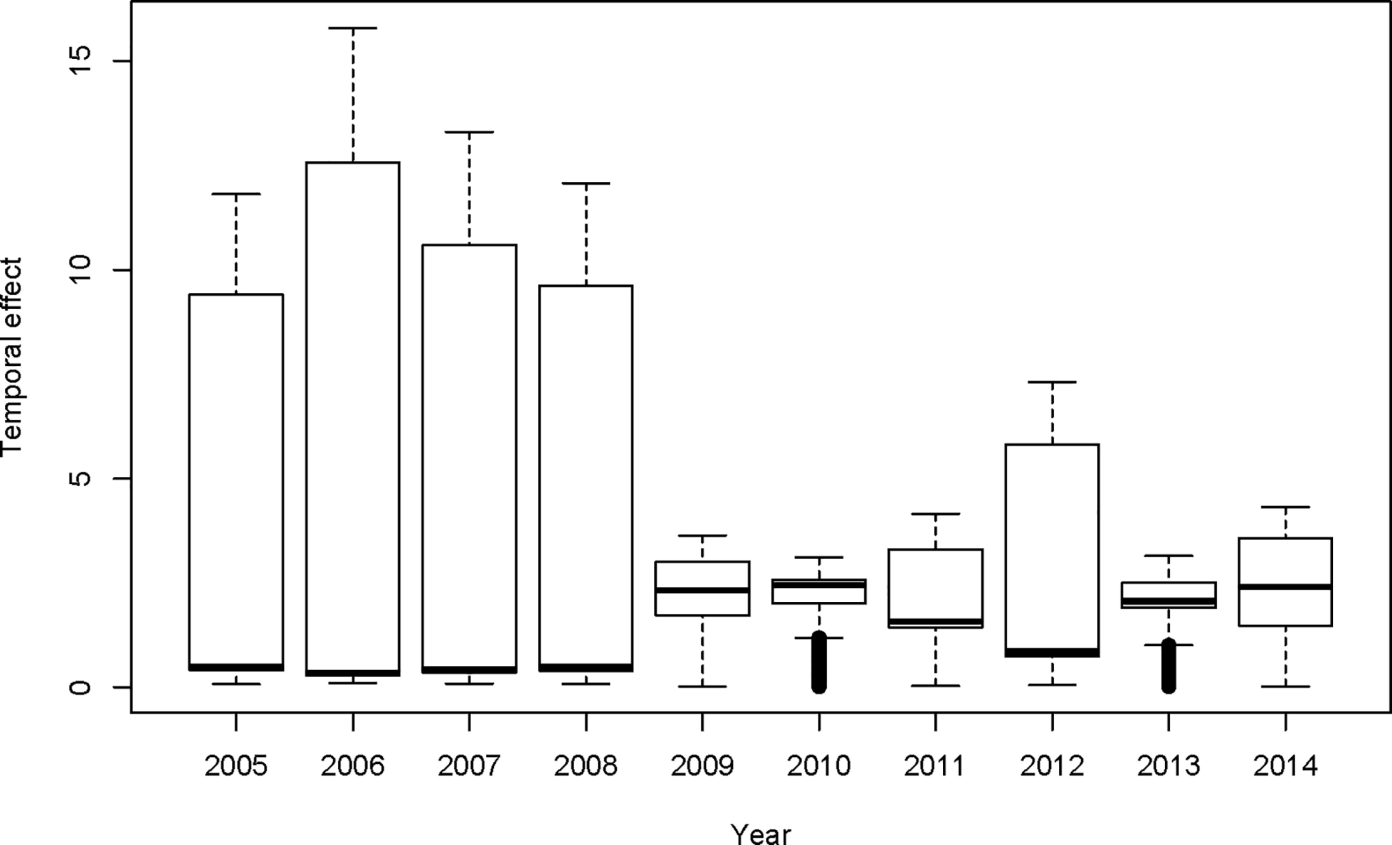
Boxplots of exponentiated posterior medians of temporal effects of measles relative risks in Namibia over the period 2005–2014.

Fig 8 shows the temporal behaviour in the measles risk in Namibia between 2005 and 2014 and it concurs with temporal trend observed in measles data before smoothing techniques were applied (Fig 2). Although there were some fluctuations in the risk of measles as the posterior means of temporal effects (when exponentiated) changed over time, it is noted that the measles risk followed an upwards trend with 2009, 2010,and 2014 as remarkable peaks in measles risk (i.e. high posterior medians in temporal effect)(Fig 7).

## Discussion

The main aim in this study was to use count data that are available at regional level for the period 2005–2014 to fit an appropriate spatio-temporal model that can be used for inference at lower level (constituency level). Thus, the study had to deal with a problem of spatial misalignment. To deal with this problem, a multi-step approach was used, which was fundamentally based on the combination of the population proportional allocation of cases for a non-uniformly distributed population and the hierarchical smoothing techniques. The results of this study are consistent with previous studies that showed spatial and temporal variability in measles risk [6, 7, 8]. The covariates used in this study, which include percentage of women aged 15–49 years with an educational level more than secondary, vaccination coverage, percentage of children aged under 5 years classified as malnourished, employment rates, and number of people aged between 0 and 24 years were only available from 2013 NDHS and 2011 NPHC. Thus, the study further aimed at correcting measurement errors in covariates asyearly data were unavailable for these covariates. It would have been restrictive to assume that covariates remained constant over time. Introducing classical measurement error models in these two covariates improved the spatio-temporal ecological regression model. Model 11, which considered measurement error models, performed better than the best model (i.e. Model 9) among the naïve models (i.e. models that ignored errors in covariates). Also, results from Model 9 indicated that “percentage of children under 5 years classified as malnourished” was not statistically significantly associated with the risk of measles (CI: -0.0609, 0.0772). However, when errors were accounted for in Model 11, this variable became significant (CI: 0.0019, 0.1128). This could be an indication that the dynamic of employment and nutrition might have changed during the period 2005–2014. In addition, this result confirmed the known fact that the common practice of not accounting for measurement error by the majority of researchers may yield misleading results [9, 10].

This study identified the number of people aged between 0 and 24 years, and the counts of measles for previous year as significant predictors of the measles risks. These findings are similar to the results of other studies conducted on other contagious diseases [6, 7, 11, 19] and demonstrated that the proxy of social mixing and the existence of pools of the disease are critical at sustaining and continuing transmission to the subsequent years in Namibia.

In this study, the percentages of women aged 15–49 years with an educational level more than secondary was found to be inversely associated with measles risk. This could be explained by the fact that educated mothers have positive attitude towards health-seeking behaviour [23, 24]. It is established that the attitude towards health seeking is one among other reasons of missing measles vaccination and underreporting of measles cases [11, 12].

As expected, we found that the vaccination coverage among of children aged 12–23 months are inversely associated with measles relative risks. It is therefore vital for Namibia to maintain existing policies (e.g. supplementary vaccination campaign every three years or in case of measles outbreaks). Furthermore, Namibia may need to improve the delivery of measles vaccines by for example borrowing and improving the standard protocol of systematic reminder/recall interventions by telephone or post, which has been proven to be an effective strategy in increasing measles vaccination coverage [25].

The study also found the employment rate and percentage of malnourished children under 5 years to be associated with measles risks. Lower employment rates are commonly associated with poor social conditions within households, which may reflect heightened close contacts of infected with susceptible vulnerable kids due to low nutritional. This finding concurs with results from the study by Kumar et al. [26].

As expected, the results showed that the constituencies in Ohangwena region were at high risk. This result is consistent with previous work by Ntirampeba *et al*. [15]. One possible explanation is that the free movement to and from Angola may enhance close contact with susceptible individuals. These findings could be useful in designing strategies and interventions such as regular border checks and targeted vaccination in high risk or along all border areas. In addition to frequent movements of populations along Namibian and Angolan borders, the high measles risk observed in Opuwo, Khorixas, and Outjo constituencies could be partly explained by low vaccination coverage (62.5%, 77.14%, and 85.2% respectively). Although Omusati region shares borders with Angola and two regions with high risk of measles (Kunene and Ohangwena), this region is among other regions that include Caprivi, Omaheke and part of Kavango found to have very low probability to be classified as areas at high risk. Further studies should be conducted to identify what could be the driving factors of low measles risk in these regions especially in Omusati, which seems to be an island among troubled areas. Furthermore, the study showed that Windhoek urban constituencies and all constituencies of Hardap had very high specific relative risks of measles.

A few limitations of this study were noted. First, the covariates were assumed to be constant over time. This assumption was dictated by the unavailability of yearly data for the covariates. Nonetheless, error measurement models used in this study may have reduced the biasedness in parameter estimates. Second, the quality and completeness of measles data could have been affected by the inequality in the distribution of health facilities across Namibia, resulting in under- reporting and miss-reporting of measles data.

Third, although the administrative boundaries of Namibia have changed over time (Kavango split into East and West Kavango in 2013), the study has used the 2011 administrative boundaries (old) because they match with variables obtained 2011 Namibia population and housing census.

## Conclusions

In conclusion, regional aggregated data were used to build a spatio-temporal model that is useful for constituency level inferences through a multi-step approach, while accounting for measurement errors in covariates. It was pointed out that there were significant variations in both spatial and temporal distribution of the measles occurrence in Namibia. Also, the study showed factors associated with measles risks in Namibia.

On the basis of the findings of this study, we recommend the following.

Firstly, the health stakeholders should increase the vaccination coverage of susceptible individuals especially in group of people aged between 0 and 24 years. Particularly, a systematical monitoring of vaccination of children aged less than five years living in poor households may help reducing the risk of measles persistence. In addition, enhancing health promotion among mothers through information, education and communication strategies should be used to improve vaccination coverage. Secondly, political leaders and stakeholders in the health sector should be able to plan and design prevention and control strategies, and make important policy decisions particularly in geographically targeted constituencies (e.g. Epupa, Kamanjab, Khorixas, Opuwo, Otjo and Sesfontein in Kunene region; and Eenhana, Endoba, Engela in Ohangwena region). Regular surveillance of population movement may assist in controlling the risk of the disease, mainly regular border checks and targeted vaccination of children in the areas identified as high risk or along all border areas.

## Supporting information

S1 Dataset Data used in this study. (XLS)

S1 Source Code R-codes used to fit Spatio-Temporal models. (TXT)

## Abbreviations

CAR: Conditional Autoregressive
CI: Credible Interval
CPO: Conditional Predictive Ordinate
NDHS: Namibia Demographic and Health Survey
DIC: Deviance Information Criterion
INLA: Integrated Nested Laplace Approximation
NSA: Namibia Statistics Agency
NPHC: Namibia population and housing census
MoHSS: Ministry of Health and Social Services
PAHO: Pan American Health Organisation
SRR: specific relative risk
WHO: World Health Organization

## References

[1] Bhella D, Bourhis JM, Rabourdin-Combe C, Cortay JC, Gahnnam A, Gerlier D, et al Measles virus nucleoprotein New York, Nova Science Publishers; 2007.

[2] Heymann DL (ed). Control of communicable diseases Washington, DC; Alpha press; 2015.

[3] WHO. WHO warns that progress towards eliminating measles has stalled. Media centre 2014. www.who.int/mediacentere/news/release/2014/eliminating-measles/en/ (2014).

[4] WHO. Measles. Fact sheet NO 286 (2015). www.who.int/mediacentre/factsheets/fs28/en.

[5] WHO. Reported measles cases and incidence rates by WHO member states 2013, 2014 as of 11February 2015. www.who.int/immunization/…/measlesreportedcasesbycountry.pdf (2015).

[6] Chiogna M, Gaetan C. Hierarchical space-time modelling of epidemic dynamics: an application to measles outbreaks. Statistical methods and Applications. 2004 4 1;13(1):55–71.

[7] Zhu Y, Xu Q, Lin H, Yue D, Song L. Spatiotemporal Analysis of Infant Measles Using Population Attributable Risk in Shandong Province, 1999–2008. PLoS ONE. 2013;8(11):e79334 10.1371/journal.pone.0079334 24260199 PMC3833981

[8] Finley AO, Banerjee S, Cook BD. Bayesian hierarchical models for spatially misaligned data in R. Methods in Ecology and Evolution. 2014;5(6):514–23.

[9] Buonaccorsi J P. Measurement error models, methods, and applications New York, Chapmann&Hall/CRC; 2010.

[10] Wattanasaruch P, Pongsapukdee V, Khawsithiwong P. Least-MSE calibration procedures for corrections of measurement and misclassification errors in generalized linear models. Songklanakarin Journal of Science and Technology. 2012;34(4).

[11] Doungmo Goufo EF, Oukouomi Noutchie SC, Mugisha S. A fractional SEIR epidemic model for spatial and temporal spread of measles in metapopulations In Abstract and Applied Analysis 2014; 22 (Vol. 2014). Hindawi Publishing Corporation.

[12] Jasem J, Marof K, Nawar A, Islam KM. Epidemiological analysis of measles and evaluation of measles surveillance system performance in Iraq, 2005–2010. International Journal of Infectious Diseases. 2012;16(3):e166–71. 10.1016/j.ijid.2011.11.002 22192582

[13] Beyene BB, Tegegne AA, Wayessa DJ, Enqueselassie F. National measles surveillance data analysis, 2005 to 2009, Ethiopia. Journal of Public Health and Epidemiology. 2016;8(3):27–37.

[14] Gotway CA, Young LJ. Combining incompatible spatial data. Journal of the American Statistical Association, 2002; 97(458), 632–648.

[15] Ntirampeba D, Neema I, Kazembe LN. Modelling spatial patterns of misaligned disease data: An application on measles incidence in Namibia. Clinical Epidemiology and Global Health. 2017;5(4):190–195

[16] Besag J, Green PJ. Spatial statistics and Bayesian computation. Journal of the Royal Statistical Society. Series B (Methodological). 1993;25–37.

[17] Dwyer-Lindgren L, et al Estimation of district-level under-5 mortality in Zambia using birth history data, 1980–2010. Spatial and spatio-temporal Epidemiology 2014; 11:89–107. 10.1016/j.sste.2014.09.002 25457599

[18] Blangiardo M, Cameletti M, Baio G, Rue H. Spatial and spatio-temporal models with R-INLA. Spatial and spatio-temporal epidemiology. 2013;7:39–55. 24377114 10.1016/j.sste.2013.07.003

[19] Restrepo AC, Baker P, Clements AC. National spatial and temporal patterns of notified dengue cases, Colombia 2007–2010. Tropical Medicine & International Health. 2014;19(7):863–71. 24862214 10.1111/tmi.12325

[20] Blangiardo M and Cameletti M. Spatial and spatio-temporal Bayesian models with R-INLA UK, John Wiley & Sons; 2015.

[21] Lawson A B. Bayesian disease mapping Hierarchical modelling in spatial epidemiology. Charleston (U.S.A), Chapmann & Hall/CRC; 2009

[22] Gustafson JP, Measurement error and missclassification in Statistics and Epidemiology: impacts and Bayesian adjustment New York, Chapmann & Hall/CRC; 2004.

[23] Adika VO, Baralate S, Agada JJ, Nneoma N. Mothers perceived cause and health seeking behaviour of childhood measles in Bayelsa Nigeria. Journal of Research in Nursing and Midwifery. 2013;2(1):6–12.

[24] Brownwright TK, Dodson ZM, van Panhuis WG. Spatial clustering of measles vaccination coverage among children in sub-Saharan Africa. BMC Public Health 2017; 17:957 10.1186/s12889-017-4961-9 29246217 PMC5732449

[25] Filia A, Bella A, Rota MC, Tavilla A, Magurano F, Baggieri M, et al Analysis of national measles surveillance data in Italy from October 2010 to December 2011 and priorities for reaching the 2015 measles elimination goal. Korea (in 2006). 2013;1:4–5. 23725868

[26] Kumar V, Chaudhury LP, Rathore R, Taneja DK, Ramnath R, Bhushan B. an epidemiological analysis of outbreak of measles in a medical relief camp. Health and Population Prespectives and Issues. 2003; 26(4):135–140.

